# Healthcare Provider Feedback Improves Outpatient E/M Billing and Coding in Otolaryngology Clinics

**DOI:** 10.1002/oto2.20

**Published:** 2023-02-26

**Authors:** Amarbir S. Gill, Dennis Menjivar, Paige Shipman, Jorgen Sumsion, Marc Error, Jeremiah A. Alt

**Affiliations:** ^1^ Division of Otolaryngology–Head and Neck Surgery, Department of Surgery University of Utah Salt Lake City Utah USA; ^2^ Department of Otolaryngology–Head and Neck Surgery University of Michigan Ann Arbor Michigan USA

**Keywords:** billing, coding, education, feedback, otolaryngology, quality improvement

## Abstract

**Objective:**

Discrepancies in medical coding can negatively impact institutional revenue and result in accusations of medical fraud. The objective of the present study was to prospectively assess the utility of a dynamic feedback system for otolaryngology providers in improving the coding/billing accuracy of outpatient clinic encounters.

**Methods:**

A billing audit of outpatient clinic visits was performed. Dynamic billing/coding feedback, consisting of a virtual lecture and targeted e‐mails, was provided at distinct intervals by the institutional billing and coding department. *χ* ^2^ was used for categorical data, and the Wilcoxon test was used to compare changes in accuracy over time.

**Results:**

A total of 176 clinic encounters were reviewed. Prior to feedback, 60% of encounters were inaccurately billed by otolaryngology providers, requiring upcoding and representing a potential 35% work relative value unit (wRVU) loss of E/M generated productivity. After 1 year of feedback, providers significantly increased the accuracy of their billing from 40% to 70% (odds ratio [OR]: 3.55, *p* < .001, 95% confidence interval [CI]: 1.69, 7.29), with a corresponding decrease in potential wRVU loss from 35% to 10% (OR: 4.87, *p* < .001, 95% CI: 0.81, 10.51).

**Discussion:**

Dynamic billing feedback significantly improved outpatient E/M coding among otolaryngology healthcare providers in this study.

**Implications for Practice:**

This study demonstrates that educating providers on appropriate medical coding and billing policies, while providing dynamic, intermittent feedback, may improve billing accuracy, translating into appropriate charges and reimbursements for services provided.

The American Medical Association published the first edition of the Current Procedural Terminology (CPT) in 1966.^1^ These codes provided a universal language of medical coding and billing for healthcare providers to use. With the advent of the electronic healthcare record, it has become increasingly important to bill for a medical visit or procedure as accurately as possible. Discrepancies in coding can have drastic consequences, including loss of revenue for healthcare institutions and possible accusations of medical fraud against a provider or facility, resulting in costly audits and fines.^2^

It is important to note that the new 2021 Medicare Evaluation and Management (E/M) guidelines have pivoted away from face‐to‐face time and/or incorporation of key components of the history and physical. Instead, these guidelines base billing and coding on the medical decision‐making (MDM) level or total time spent on the date of the encounter.^3^ Aiming to reduce the documentation burden for physicians, this change was made in parallel with a recent revaluing of services rendered, resulting in an increase in work relative value units (wRVUs) for E/M codes.

Although data on the accuracy of medical coding by providers in the outpatient clinic setting is limited, studies across a few non‐otolaryngologic specialities suggest the possibility of significant inaccuracy.^4^, ^5^, ^6^, ^7^ No similar studies have been performed in otolaryngology. In the present investigation, we sought to prospectively characterize the accuracy of outpatient clinic encounter billing and coding by otolaryngology providers. Further, we assessed the role of a dynamic feedback and education system for providers in improving this accuracy over time. We hypothesized that providers significantly underbilled clinic visits and that the incorporation of a feedback system would significantly improve the accuracy of clinic billing.

## Methods

The International Review Board (IRB) at the University of Utah determined the present investigation does not meet the definitions of Human Subjects Research according to Federal regulations and, as such, did not require IRB oversight. An audit of outpatient clinic visits was performed at distinct intervals over the year 2021 by the billing and coding department at the University of Utah, after the introduction of the 2021 Medicare Evaluation and Management (E/M) guidelines, in order to provide feedback to improve coding accuracy.

The CPT codes reviewed in the present investigation included 99202 to 99205 (for established patients) and 99212 to 99215 (for new patient visits). The most basic of these codes are 99202/99212 and increase incrementally to 99205/99215 based on the complexity of the visit or as a result of the total time spent on the day of the encounter, according to the E/M guidelines.^3^ An initial virtual lecture on the new 2021 E/M system was given to all otolaryngology providers by the institution's billing and coding department in January 2021. This lecture outlined the 2021 E/M billing criteria and highlighted how they differed from the prior guidelines. The subsequent, dynamic billing feedback consisted of interval electronic correspondence (e‐mails, see Supplemental Figure 1, available online, for templated example) (March 2021 and June 2021) from the same institutional billing and coding department, providing feedback and education to providers on inaccuracies identified, and how to correct these inaccuracies for each provider. The feedback highlighted the most common codes being billed incorrectly based on the billing and coding audits of those codes and explained the reasoning behind alternative codes being better options.

Based on these three interventions, we divided our study into 3 distinct periods. Interval 1 began in January 2021 and extended to March 2021, which was between the initial virtual lecture on the billing system and the first feedback email. Interval 2 went from April 2021 to June 2021, encompassing the period of time between the first and second feedback email to providers. Interval 3 (July 2021 to December 2021) comprised the period of time between the second audit email and the end of the year.

Billing inaccuracies among audited encounters from five different otolaryngology providers were characterized for each aforementioned CPT code and interval. We used 2021 Medicare wRVU assignments for each CPT code (Supplemental Table 1, available online) to determine whether wRVU gained or lost due to inaccuracies identified and rectified.^8^ *χ* ^2^ was used for categorical data, and the Wilcoxon test was used to compare the change in accuracy over time. Effect sizes and 95% confidence intervals (CI) were calculated, and the threshold for significance was set at *p* < .05.

## Results

### Impact of Feedback/Education on Billing/Coding Accuracy

**Figure 1:**
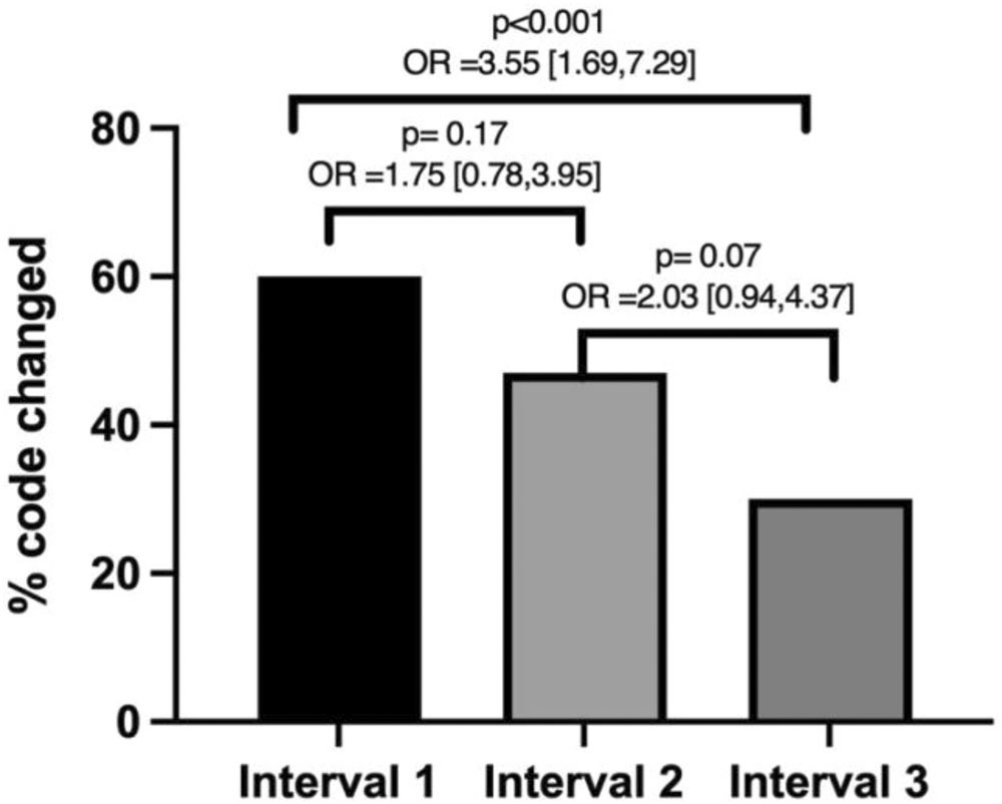
Change in billing and coding over time. OR, odds ratio.

A total of 176 encounters were audited over the course of the study. During interval 1 (ie, after the initial virtual lecture on the new 2021 E/M guidelines but before any interval feedback emails), 32/53 (60%) billing codes required changing (all 32 were upcoded) (Table 1). The number of codes requiring change decreased over the course of the study year. During interval 2, 20/43 (47%) codes needed to change (all 20 were upcoded), while in interval 3, 24/80 (30%) needed to be changed (all except 1 of the 24 were upcoded) (Table 1). The improvement in the accuracy of billing from interval 1 to interval 3 was statistically significant (*p* < .001) with an odds ratio of 3.55 and 95% CI of (1.69, 7.29) (Figure 1). The individual codes that needed to be changed during each interval are outlined in Supplemental Tables 2a‐c, available online.

**Table 1:** Billing/Coding Audit Over the Study Year

Clinic Encounters—Billing/coding	Interval 1	Interval 2	Interval 3
Changed	32 (60%)	20 (47%)	24 (30%)
Accurate	19 (40%)	23 (53%)	56 (70%)
Total reviewed	53	43	80

### Impact of Feedback/Education on wRVUs

**Figure 2:**
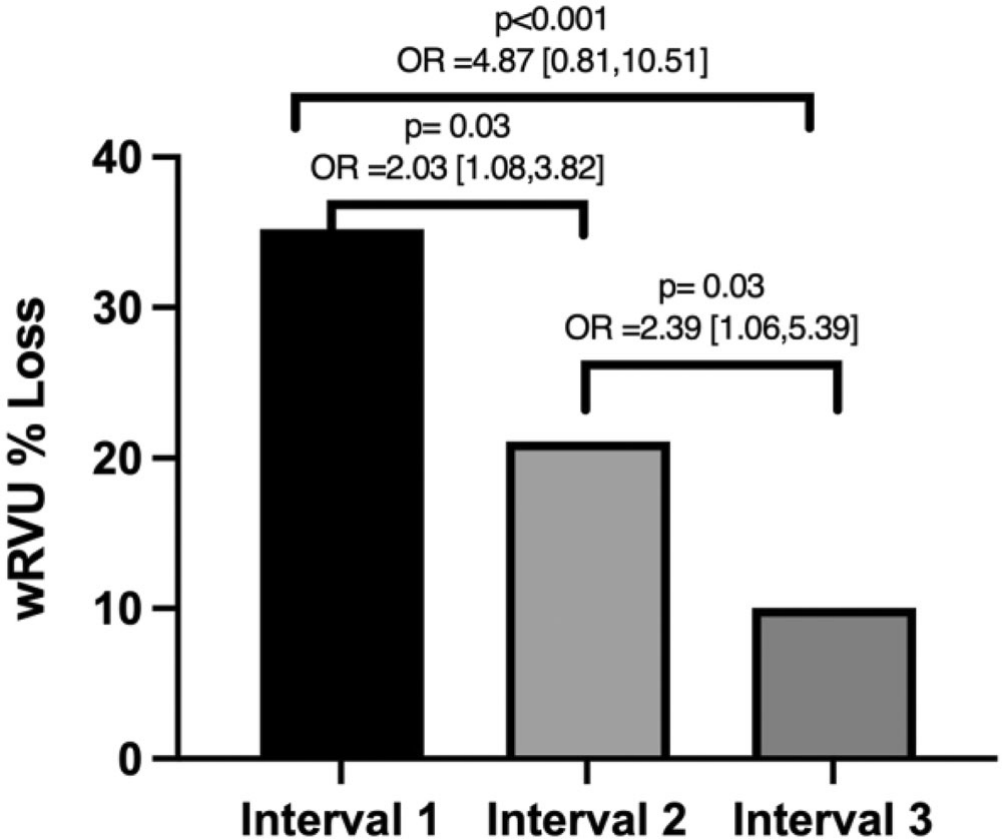
Change in wRVU loss over time. OR, odds ratio; wRVU, work relative value unit.

There was a potential loss of a total of 60.33 wRVUs based on the changes in billing/coding that were performed on the 176 audited encounters (Table 2). This potential wRVU loss represented 19.2% of the total wRVU generated from clinic E/M coding during the entire study period (Table 2). Overall, the wRVU loss decreased over time from 35.2% of the total in interval 1 to 21.1% of the total in interval 2 (*p* = .03, odds ratio [OR]: 2.03, 95% CI: 1.13‐4.54) and 10.04% of the total in interval 3 (*p* < .001, OR: 4.87, 95% CI: 0.96, 3.68) (Table 2, Figure 2). Of note, Supplemental Tables 1 and 2, available online, detail the individual time intervals' audits and corresponding changes to wRVUs.

**Table 2:** Change in wRVU Loss Over the Study Year

	Interval 1	Interval 2	Interval 3
wRVU loss (%)	35.2	21.1	10.04
Total codes reviewed	53	43	80

Abbreviation: wRVU, work relative value unit.

### Feedback/Education on Billing Elements Used by Providers

Over the course of the study, otolaryngology providers increased the incorporation of MDM and time as billing elements, as opposed to relying purely on MDM. In interval 1, 46/53 encounters were billed only on the basis of MDM, while only 5/53 used both MDM and time. In interval 2, MDM was used in 34/43 instances, with 9/43 encounters utilizing both MDM and time. In interval 3, MDM was used in 48/80 encounters, with 32/80 encounters now utilizing both MDM and time. Ultimately, there was a significant increase in the incorporation of both MDM and time from interval 1 to interval 3 (*p* < .001, OR 0.16, 95% CI: 0.06, 0.43) (Supplemental Figure 2, available online). Of note, Supplemental Table 3, available online, outlines the billing elements used by providers during each interval.

## Discussion

The present investigation demonstrated a significant baseline level of inaccurate billing and coding within a tertiary care otolaryngology program after the implementation of the 2021 E/M guidelines. Nevertheless, the implementation of a dynamic feedback mechanism, with provider education regarding medical coding and billing, significantly improved accuracy over the course of a year. Of note, when discrepancies arose, visits were overwhelmingly more likely to be underbilled (ie, 75/76 encounters) than overbilled (ie, 1/76 encounters). Ultimately, improvement in E/M coding over the time period of the study significantly reduced underbilling and potential wRVU loss from 35% to 10%.

Despite increasing pressures on healthcare providers to correctly code and document, there is no set curriculum in residency or beyond to educate physicians in correctly coding and documenting clinical encounters. Although restricted in its scope, the literature demonstrates a poor fund of knowledge regarding clinic billing/coding among trainees, with the potential for significant underbilling.^4^, ^5^, ^9^ Limited, often single instance, educational interventions with a lecture have resulted in improvements in coding/billing accuracy across various subspecialties among trainees.^4^, ^5^, ^10^ The few studies assessing the role of such limited billing/coding interventions on non‐trainee healthcare providers have incorporated clinical scenarios, rather than analyzed actual billing/coding data from clinic visits.^10^

Indeed, there is limited data on the role of various interventions in decreasing revenue loss in healthcare in general. Nevertheless, when implemented correctly, interval, dynamic feedback in nonclinic scenarios, such as in the operating room, has been shown to decrease revenue loss.^11^, ^12^, ^13^ With this in mind, our group sought to understand the role of a feedback mechanism aimed at improving clinic billing/coding among otolaryngology providers. We demonstrated sustainable improvements in billing and coding accuracy over the course of a year with the incorporation of the interval, and dynamic feedback from billing auditors, helping address a critical knowledge gap in the existing literature.

The present investigation also sought to evaluate the frequency with which MDM versus time was implemented as the billing element. The data demonstrated that MDM was primarily used by providers to bill a clinic visit during the interval I and that the time element alone was rarely used. However, over time, providers successfully incorporated both the MDM and time element into their billing, with a concomitant decrease in the number of clinic visit encounters needing to be changed on billing audits. Ultimately, as demonstrated by Hager and Gosser,^14^ incorporation of time as part of the billing element may improve billing accuracy and reduce lost revenue.

Both overbilling and underbilling can have a negative impact both on patients receiving care as well as the healthcare institution. Overbilling can lead to denials, review by malpractice carriers, and even possible fraud or abuse charges.^15^ While many healthcare providers may feel that underbilling can help avoid these serious consequences, this does not mean that it does not also carry its share of negative effects.^15^ For example, in the year 2000, a primary care provider underbilling 99,212, which paid $40.20, instead of the more appropriate 99,213, which paid $54.68, could lead to a loss of revenue totaling $90,000 over a single year.^15^ This translates into an even greater loss of revenue with the implementation of the 2021 E/M guidelines, which demonstrates larger gaps between reimbursement for 99,212 ($36.56) and 99,213 ($93.51).^16^

There are several limitations to the present investigation that should be acknowledged. The sample size is small, although comparable to the few, similar, non‐otolaryngology investigations assessing billing accuracy in the outpatient setting. Although this study demonstrates that a dynamic feedback/education mechanism can be beneficial in improving billing accuracy, it does not demonstrate whether feedback over the course of one year is sufficient for improving billing accuracy long‐term or if continued feedback is required to maintain an improved level of accuracy. This is a single‐institution investigation that only examined otolaryngology providers, which limits its generalizability. Future studies should incorporate other medical and surgical subspecialties to better understand the widespread nature of the problem of inaccurate coding/billing among healthcare providers. It is possible that providers also obtained information on coding from other resources during this time, which could have also had an influence on billing behaviors. Given the quality improvement and pilot nature of the present investigation, we did not incorporate a control group of providers who were not provided billing feedback. Finally, the contribution of 5 separate providers to the data set introduces the possibility of clustering effects.

## Implications for Practice

The present investigation sheds light on a critical knowledge gap that exists regarding the accuracy of outpatient clinical encounters in otolaryngology and the means by which to improve current deficiencies. This study demonstrates that educating providers on appropriate medical coding and billing policies, while providing dynamic, intermittent feedback, may improve billing accuracy, translating into appropriate charges and reimbursements for services provided. Future work in this domain should consider the incorporation of a control group to help strengthen the results presented in this pilot investigation.

## Author Contributions

**Amarbir S. Gill**, conceptualization and design, data analysis, writing; **Dennis Menjivar**, data collection, writing; **Paige Shipman**, data collection, data analysis, writing; **Jorgen Sumsion**, data analysis, revisions; **Marc Error**, conceptualization and design, revisions; **Jeremiah A. Alt**, conceptualization and design, revisions.

## Disclosures

### Competing interests

J.A.A: Consultant for Medtronic, Optinose, GSK, and GlycoMira. A.S.G., D.M., P.S., J.S., and M.E.: None.

### Sponsorships

None.

### Funding source

None.

## Supporting information

Supporting information.

Supporting information.

Supporting information.

Supporting information.

Supporting information.

Supporting information.
