# Supplementary figures and images for: Healthcare Provider Feedback Improves Outpatient E/M Billing and Coding in Otolaryngology Clinics
Source: OTO Open. 2023 Feb 26;7(1):e20. doi: 10.1002/oto2.20 (PMC10046709; doi:10.1002/oto2.20)

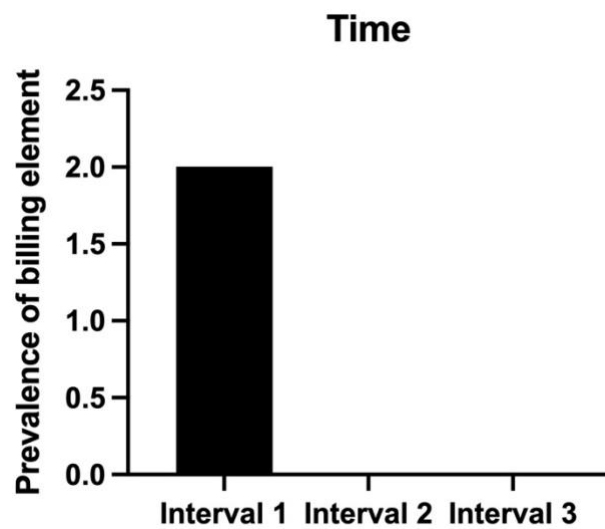

Supplement: Supplementary file 2 — Supporting information. [file OTO2-7-e20-s006.pdf]

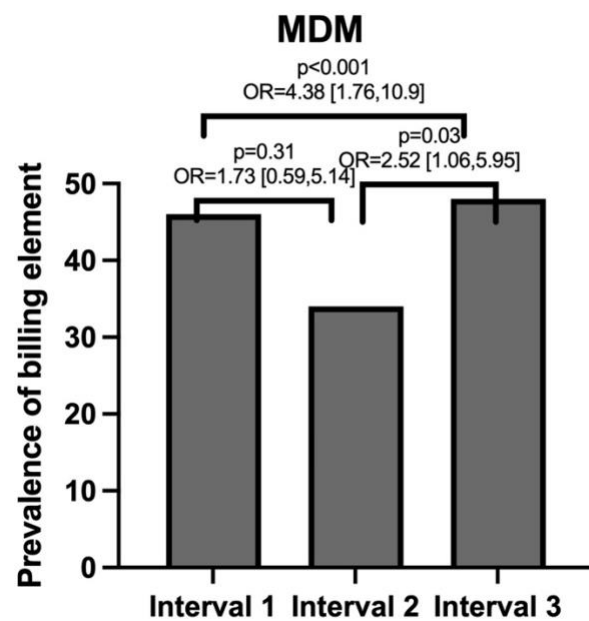

Supplement: Supplementary file 3 — Supporting information. [file OTO2-7-e20-s001.pdf]

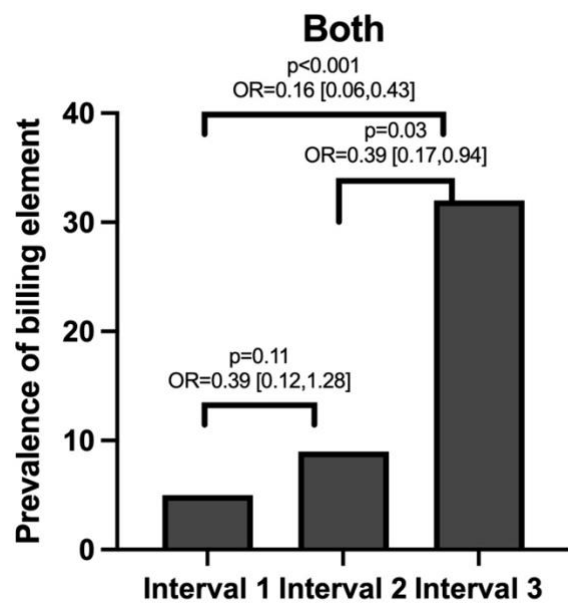

Supplement: Supplementary file 4 — Supporting information. [file OTO2-7-e20-s002.pdf]
