# Supplementary material for: Healthcare Provider Feedback Improves Outpatient E/M Billing and Coding in Otolaryngology Clinics
Source: OTO Open. 2023 Feb 26;7(1):e20. doi: 10.1002/oto2.20 (PMC10046709; doi:10.1002/oto2.20)
Supplement: Supplementary file 5 — Supporting information. [file OTO2-7-e20-s003.docx]

Supplemental Table 1

|  |  |  |  |  | |  | |  | | **Reason for change in billing** | |
| --- | --- | --- | --- | --- | --- | --- | --- | --- | --- | --- | --- |
|  | **Number Reviewed** | **wRVU** | **Number Changed** | **UPCODED 1 LVL** | **change in wRVU (%)** | **UPCODED 2 LVL** | **change in wRVU** | **DOWNCODED 1 LVL** | **change in wRVU (%)** | **Time spent** | **MDM** |
| **Interval 1** | **53** | **53.07** | **32** | **24** | **+15.52**  **(+22.6%)** | **8** | **+13.36**  **(+20.1%)** | **0** | **0** | **1** | **31** |
| **Interval 2** | **43** | **56.68** | **20** | **16** | **+9.88 (+14.8%)** | **4** | **+5.33 (+8.6%)** | **0** | **0** | **2** | **18** |
| **Interval 3** | **80** | **145.44** | **24** | **20** | **+13.48 (+9.27%)** | **3** | **+3.66 (+2.5%)** | **1** | **-0.9**  **(-0.62%)** | **9** | **15** |

Supplemental Table 2a

|  |  |  |  |  | |  | | **Reason for change in billing** | |
| --- | --- | --- | --- | --- | --- | --- | --- | --- | --- |
|  | **Number reviewed** | **wRVU** | **Number changed** | **UPCODED 1 LVL** | **change in wRVU** | **UPCODED 2 LVL** | **change in wRVU (%)** | **Time spent** | **MDM** |
| **CPT/Description** |  |  |  |  |  |  |  |  |  |
| **99202** | 1 | 0.93 | 1 | 0 | 0 | 1 | 1.67 | 0 | 1 |
| **99212** | 39 | 27.3 | 26 | 19 | 11.4 | 7 | 11.69 | 0 | 26 |
| **99203** | 2 | 3.2 | 2 | 2 | 2 | 0 | 0 | 0 | 2 |
| **99213** | 5 | 6.5 | 2 | 2 | 1.24 | 0 | 0 | 0 | 2 |
| **99204** | 3 | 7.8 | 0 | 0 | 0 | 0 | 0 | 0 | 0 |
| **99214** | 2 | 3.84 | 1 | 1 | 0.88 | 0 | 0 | 1 | 0 |
| **99205** | 1 | 3.50 | 0 | 0 | 0 | 0 | 0 | 0 | 0 |
| **99215** | 0 | 0 | 0 | 0 | 0 | 0 | 0 | 0 | 0 |
| **TOTALS** | **53** | **53.07** | **32** | **24** | **+15.52 (+22.6%)** | **8** | **+13.36 (+20.1%)** | **1** | **31** |

Supplemental Table 2b

|  |  |  |  |  | |  | | **Reason for change in billing** | |
| --- | --- | --- | --- | --- | --- | --- | --- | --- | --- |
|  | **Number Reviewed** | **wRVU** | **Number Changed** | **UPCODED 1 LVL** | **change in wRVU** | **UPCODED 2 LVL** | **change in wRVU** | **Time spent** | **MDM** |
| **CPT/Description** |  |  |  |  |  |  |  |  |  |
| **99202** | 2 | 1.86 | 1 | 0 | 0 | 1 | 1.67 | 0 | 1 |
| **99212** | 23 | 16.1 | 18 | 15 | 9 | 3 | 3.66 | 1 | 17 |
| **99203** | 1 | 1.60 | 0 | 0 | 0 | 0 | 0 | 0 | 0 |
| **99213** | 3 | 3.9 | 0 | 0 | 0 | 0 | 0 | 0 | 0 |
| **99204** | 7 | 18.2 | 0 | 0 | 0 | 0 | 0 | 0 | 0 |
| **99214** | 6 | 11.52 | 1 | 1 | 0.88 | 0 | 0 | 1 | 0 |
| **99205** | 1 | 3.50 | 0 | 0 | 0 | 0 | 0 | 0 | 0 |
| **99215** | 0 | 0 | 0 | 0 | 0 | 0 | 0 | 0 | 0 |
| **TOTALS** | **43** | **56.68** | **20** | **16** | **+9.88 (+14.8%)** | **4** | **+5.33 (+8.6%)** | **2** | **18** |

Supplemental Table 2c

|  |  |  |  |  | |  | |  | | **Reason for change in billing** | |
| --- | --- | --- | --- | --- | --- | --- | --- | --- | --- | --- | --- |
|  | **Number Reviewed** | **wRVU** | **Number Changed** | **UPCODED 1 LVL** | **change in wRVU** | **UPCODED 2 LVL** | **change in wRVU** | **DOWNCODED 1 LVL** | **change in wRVU (%)** | **Time spent** | **MDM** |
| **CPT/Description** |  |  |  |  |  |  |  |  |  |  |  |
| **99202** | 0 | 0 | 0 | 0 | 0 | 0 | 0 | 0 | 0 | 0 | 0 |
| **99212** | 20 | 14 | 15 | 12 | 7.2 | 3 | 3.66 | 0 |  | 2 | 13 |
| **99203** | 2 | 3.2 | 0 | 0 | 0 | 0 | 0 | 0 | 0 | 0 | 0 |
| **99213** | 10 | 13 | 1 | 1 |  | 0 | 0 | 0 | 0 | 0 | 1 |
| **99204** | 20 | 52 | 6 | 6 | 5.4 | 0 | 0 | 0 | 0 | 6 | 0 |
| **99214** | 22 | 42.24 | 1 | 1 | 0.88 | 0 | 0 | 0 | 0 | 1 | 0 |
| **99205** | 6 | 21 | 1 | 0 | 0 | 0 | 0 | 1 | -0.9 (-25.7) | 0 | 1 |
| **99215** | 0 | 0 | 0 | 0 | 0 | 0 | 0 | 0 | 0 | 0 | 0 |
| **TOTALS** | **80** | **145.44** | **24** | **20** | **+13.48 (+9.27%)** | **3** | **+3.66 (+2.5%)** | **1** | **-0.9 (-0.62%)** | **9** | **15** |

Supplemental Table 3

| **BILLING ELEMENTS USED BY PROVIDER** | | | | |
| --- | --- | --- | --- | --- |
|  | **TIME ONLY** | **MDM ONLY** | **BOTH*** | **TOTAL** |
| **Interval 1** | **2** | **46** | **5** | **53** |
| **Interval 2** | **0** | **34** | **9** | **43** |
| **Interval 3** | **0** | **48** | **32** | **80** |
