# Supplementary material for: Healthcare Provider Feedback Improves Outpatient E/M Billing and Coding in Otolaryngology Clinics
Source: OTO Open. 2023 Feb 26;7(1):e20. doi: 10.1002/oto2.20 (PMC10046709; doi:10.1002/oto2.20)
Supplement: Supplementary file 6 — Supporting information. [file OTO2-7-e20-s004.docx]

**Supplemental Figure 1:** Sample template of feedback provided by billing professionals to providers on review of billing/coding accuracy and areas for improvement

*MDM criteria can be found at: https://www.ama-assn.org/system/files/2019-06/cpt-office-prolonged-svs-code-changes.pdf^3^

**Supplemental Figure 2a-c:** Changes in billing element incorporation over time

1. Prevalence of using time as the only billing element during the study year
2. Prevalence of using medical decision making (MDM) as the only billing element during the study year
3. Prevalence of using BOTH time and medical decision making (MDM) as the billing elements during the study year

**Supplemental Table Legends**

**Supplemental Table 1:** Overall billing changes made during time intervals 1-3. CPT: current procedural terminology; wRVU: work relative value unit; LVL: level; MDM: medical decision making

**Supplemental Table 2a:** Billing changes by individual CPT codes during time interval 1. CPT: current procedural terminology; wRVU: work relative value unit; LVL: level; MDM: medical decision making

**Supplemental Table 2b:** Billing changes by individual CPT codes during time interval 2. CPT: current procedural terminology; wRVU: work relative value unit; LVL: level; MDM: medical decision making

**Supplemental Table 2c:** Billing changes by individual CPT codes during time interval 3. CPT: current procedural terminology; wRVU: work relative value unit; LVL: level; MDM: medical decision making

**Supplemental Table 3**: Prevalence of documenting time vs MDM vs both, time intervals 1-3. MDM: medical decision making.

*Documenting both time and MDM.
