# Supplementary material for: Healthcare Provider Feedback Improves Outpatient E/M Billing and Coding in Otolaryngology Clinics
Source: OTO Open. 2023 Feb 26;7(1):e20. doi: 10.1002/oto2.20 (PMC10046709; doi:10.1002/oto2.20)
Supplement: Supplementary file 1 — Supporting information. [file OTO2-7-e20-s005.pdf]

Dear Dr. X,

Please see below comments highlighting areas for improvement in billing/coding in _ number of audited outpatient clinic encounters. The percentage of opportunity for improvement on outpatient E/M billing was noted to be _%.

Summary: Time was only reported in _% of encounters, which could impact coding for level of service, especially for prolonged service codes. _% of outpatient E/M codes were changed to higher level codes. The majority of lower-level codes billed were due to lack of identified E/M criteria. Thus, for your consideration, we have provided information below on items that may have been performed but not documented.

New/Established Encounters Audited (trends noted)

- A total of _ records were reviewed
- _% E/M codes were changed (_% were changed to a higher code, _% were changed to a lower code)
- The most frequent code changed was _ and it was most commonly changed to _
- Time was not consistently documented
- Documentation did not support individual interpretation of items such as radiology, audiograms, etc
- Documentation did not support review of records

Billing and coding education opportunities

- Medical decision making (MDM) criteria*
  - I. List the number and complexity of problems being addressed during the clinic encounter
    - a. Document chronic conditions
    - b. Document disease progression (exacerbation, chronicity, resolution, side effects or complications)
  - II. Outline the data being analyzed/reviewed
    - a. Document each test ordered/reviewed
    - b. Document each source accessed for external review
    - c. Document independent interpretation of diagnostic testing (radiology, audiogram, etc)
  - III. Communicate risks (morbidity/mortality/complications) of patient management decisions during visit for each problem addressed
    - a. Discussion/decision surrounding diagnostic testing, treatment, surgery, hospitalization, etc
    - b. Prescription drug management
    - c. Management impacted by social determinants
- Outpatient time
  - I. Document total time spent on the date of the billing encounter.

a. You cannot utilize time as a part of your E/M service if time/note is not done on the same day as the visit.
b. You cannot include time spent by a resident, fellow, clinical staff toward billable time
c. For physician and advance practice providers, only one individual can be counted per minute and both providers should document their own time. Total time should be based on the combination of both providers.

Level	New	Established
2	15 – 29 min	10 – 19 min
3	30 – 44 min	20 – 29 min
4	45 – 59 min	30 – 39 min
5	60 – 74 min	40 – 54 min
